# The role of double-skin facade configurations in optimizing building energy performance in Erbil city

**DOI:** 10.1038/s41598-023-35555-0

**Published:** 2023-05-24

**Authors:** Mohammed Siyamand Naddaf, Salahaddin Yasin Baper

**Affiliations:** Architectural Engineering Department, Salahaddin University, Erbil, 44001 Iraq

**Keywords:** Engineering, Energy science and technology

## Abstract

Carefully designing a building facade is the most crucial way to save energy, and a double-skin facade is an effective strategy for achieving energy efficiency. The improvement that can be made depends on how the double-skin facade is set up and what the weather conditions are like. This study was designed to investigate the best-case scenario with an appropriate double-skin facade configuration for optimizing building energy performance. A methodology for optimizing the building's initial condition was introduced using EnergyPlus and ClimateStudio according to a 1-year period of the city of Erbil. Analysis of double-skin parameters was performed by utilizing a multi-objective analysis approach. Four naturally ventilated geometric configurations were assessed: building-height, storey-height, shaft-box, and box-window. The results provide annual and seasonal consumption curves for each orientation. The massive airflow between adjacent thermal zones of a shaft-box facade significantly reduces the amount of cooling energy needed. Hence, due to the intricate internal partitioning that allows for airflow within the cavity and shaft, this design indicates multiple advantages over others. The annual cooling demand drops significantly, by 9% to 14%. Energy savings of up to 116,574 kWh per year are possible when using a double-skin facade compared to the building’s initial condition, which is a great asset in the temperate environment of Erbil.

## Introduction

The exterior of a building, known as the facade, plays a major role in connecting indoor and outdoor environments and highly affect the temperature inside the building and energy usage. High-performance facade systems involve selecting and implementing the right materials, cutting-edge technologies, proper detailing and installation appropriate for the specific context and function. A well-designed facade can protect a building from summer heat, minimize heat loss during winter, and utilize natural elements for heating, cooling and lighting. In recent times, building facades, particularly glazing systems, have been widely researched and developed due to their ability to improve energy efficiency and reduce the impact of buildings on the environment^1^.

A double-skin facade (DSF) is a multi-layered skin that was originally designed for the cold climates of European countries, with notable success at the beginning of the twenty-first century^2^. Over the past decade, many buildings with double-skin facades have been constructed, featuring different variations in the technology^3–5^. To evaluate their performance over time, a field of academic research has emerged to study the long-term performance of these installations. Since then, to prevent any unexpected issues in real life, countries with harsh climates have been investigating the idea of implementing DSFs through the use of computer simulations. Heat gain or loss through the building envelope accounts for 20 to 50 percent of the total energy used by air-conditioning systems, indoor heating, and ventilation^6^.

Through years of research and ongoing improvement, the DSF system has evolved into its current form and has come to be associated with applications of transparent and glass architecture. It is also evolving into a successful environmental design approach for reducing energy consumption and life cycle costs^7^. DSFs are becoming increasingly significant in contemporary building practice that reduces wind speed and noise^4^. Even while summertime overheating of double facades is inevitable, it can be reduced with shading devices, well-designed openings, and an optimized air gap between skins^8^.

During cooling and heating periods, a DSF operates differently. In hot climates, heat accumulates in the cavity and is partially transferred to the adjacent space via air introduced through the cavity openings. The stack effect moves excess heat to the outside of a building. Differences in air density cause a circular flow that releases hotter air, lowering the temperature of the DSF's inner layer and reducing the amount of heat transferred into the interior space. Finally, as the temperature rises in the air cavity, the pressure is released upwards, creating a light breeze and minimizing heat gain, reducing the cooling demand of the occupied space^9^.

The thermal performance of DSFs is highly dependent on weather conditions; however, tests of various models and their variables in diverse climates indicate that DSFs could potentially improve energy performance^2^. A primary objective for reducing energy consumption and the environmental impact of the construction sector is increasing the energy efficiency of current public buildings^10^. However, it is unclear which configuration of a DSF can produce the best retrofit in hot climates when utilized on different facade orientations. Determining the best-case solution with the proper DSF setup to maximize building energy performance was the aim of the current study. Some applications in warmer climates have been studied and produced unfavourable performance, but those studies also identified how design and operation affected the overall thermal performance of buildings^11^. In addition, the advantages and disadvantages have been examined by researchers^12^.

DSFs can provide the increased facade transparency that is highly valued in today's society, but they can also offer new opportunities for architectural design by altering, moving, and separating traditional architectural elements^13^. The thermal performance of a DSF highly depends on its design and local context, such as climate conditions^14^. However, the following inquiries are of interest: Which DSF configuration provides the best energy performance in Erbil buildings during the summer? Which DSF parameters affect the efficiency and reliability, as well as the energy performance, of buildings in such climate conditions?

## Literature review

### Related studies on DSFs and their roles in energy performance

Many studies consistently indicate that a DSF is more cost-effective in the long run because it is longer lasting and more durable than a single-skin facade (SSF)^7,15,16^. It creates a more comfortable and environmentally friendly environment and lowers maintenance costs by conserving the building's energy resources^16^. Furthermore, for life cycle assessment analysis, despite having a higher initial energy and material cost, the DSF system has the potential to decrease annual energy cost and CO_2_ emissions by 9.2%^7^.

The orientation and climate have a significant impact on how the sealed cavity facades and open joint ventilated facades behave. The rate of energy savings rises with solar and external air temperatures^17^. Orientation and WWR are correlated, when a small modification in the window’s aperture has a big impact on the energy efficiency and operating costs. Lighting, heating, and cooling energy demands can all be altered using WWR optimization. The window sill, and the window's position in relation to the facade have no impact on EUI^18^.

Significant potential as an appropriate energy efficiency solution for the building sector in the middle eastern region is highlighted, along with quantitative outputs for lowering annual cooling and thermal loads, increasing grid-connected electricity generation, and improving the energy performance index of existing buildings with the use of DSF photovoltaic thermal system^19^.

Systematic comparison with 15 climate types (totalling 150 energy models) concentrating on energy performance, assessing energy consumption in a range of temperature climate types. Although energy savings for heating and cooling were examined across different DSF types, the study was unable to confirm the impact of DSF designs on energy usage^20^. A numerical model for multiple-skin facades with mechanical and natural ventilation has been developed in hot arid climate, most typologies cannot simultaneously lower cooling demand, but by combining typologies or adjusting the system to the condition, a significant improvement can be achieved^21^. However, there has been no systematic assessment of the thermal and energy performance of DSFs in all configurations. To address this point, the (Table 1) summarizes the available literature and past study findings that classified DSFs based on building parameters, climate conditions, and economic considerations.

**Table 1:** Summary of previous related studies and their key findings.

	Study	Parameters of the experiment		Main finding
DSF building parameters	^22^	Variables	BES, base model	The built-in model was the most reliable predictor of the chosen parameters in two out of the three tools when two approaches were evaluated against experiment data
		Tools	EnergyPlus, TRANSYS	
		Config	SH*	
		Mode	Mechanical ventilation	
	^17^	Variables	Glazing	In comparison to naturally ventilated DSFs with outside double glass, naturally ventilated DSFs with interior double glass perform thermally better
		Tools	MATLAB	
		Config	SH*	
		Mode	Natural ventilation	
	^8^	Variables	Cavity width	When two configurations are combined, the advantages of DSFs is evident, especially at lower airflows (15 m^3^/h and 50 m^3^/h), where a large temperature increase (up to 15.9 °C) occurs
		Tools	Ansys fluent	
		Config	BH, SH, SB, BW*	
		Mode	Natural ventilation	
	^20,23^	Variables	U-value, climate zone, EUI	The thermal performance of the various DSF kinds varies little, but all DSF facades provide greatly increased thermal performance compared to the baseline SSF
		Tools	DesignBuilder	
		Config	BH, SH, BW*	
		Mode	Mechanical ventilation	
	^24^	Variables	Cavity width, glazing materials, orientation	Because of the reduced solar gain by the exterior facade, DSFs can lower cooling loads whether the channel is vented or not. Supplying natural ventilation to the cavity accelerates the heat change when the volume is sufficient for the airflow to impinge on the inner skin surfaces
		Tools	IES-virtual Environment	
		Config	SH*	
		Mode	Natural ventilation	
	^4^	Variables	South orientation	A DSF reduces losses caused by transmission of interior facades and protects them from losses caused by infrared radiation
		Tools	TAS	
		Config	SH*	
		Mode	Mechanical ventilation	
	^25,26^	Variables	Green facade, orientation, material, WWR, cavity	The results shows that DSF with greenery were more energy-efficient in both climates and by using different materials for the DSF (brick, wood, and metal pane). For all opaque materials, EUI decreased when WWR increased, and increased when the cavity width increased
		Tools	DesignBuilder, EnergyPlus	
		Config	SH*	
		Mode	Supply/natural ventilation	
DSF climate consideration	^9^	Variables	Orientation, energy consumption	By reducing energy use, DSFs in Mediterranean climate office buildings can save up to 299,279 kWh annually. Energy consumption was lowered by 28% in the winter and 53.5% in the summer when the model was constructed with three DSFs on the east, south, and west facades
		Tools	Ecotect	
		Config	BH*	
		Mode	Natural ventilation	
	^27,28^	Variables	WWR, material, Orientation	In contrast to the SSF model, the results showed that it was feasible to build an energy-saving DSF system for use in hot and humid conditions. In hot summer days reduces cooling energy usage possible by approximately 0.27 kWh/m^2^ every day, saving electricity and 159 g-CO_2_/m^2^
		Tools	DesignBuilder	
		Config	SH*	
		Mode	Natural ventilation	
	^11,29^	Variables	Cavity width	In hot, humid climates, a DSF enables a potential 22% reduction in the annual cooling energy consumption compared to the baseline. The annual cooling energy consumption of the building might be decreased by 32% by adding mechanical ventilation to the air cavity
		Tools	DesignBuilder, EnergyPlus	
		Config	BH, SH, SB, BW*	
		Mode	Mechanical ventilation	
	^2^	Variables	Comparison	The outcomes of case studies from various climate zones are compared. The findings also demonstrate how significantly the thermal performance of DSF is enhanced by skin material, blind devices, and optimum ventilation strategy
		Tools	IES-VE, Ansys fluent	
		Config	BH, BW*	
		Mode	All modes	
Economy consideration	^7^	Variables	Life cycle, South, West	Despite having a higher embodied energy and material cost of a DSF system, LCA suggests that it might lower annual operational energy and CO_2_ emissions by 9.2%
		Tools	Experimental	
		Config	SB*	
		Mode	Natural ventilation	
	^15,30^	Variables	Energy consumption	The DSF has a longer-term cost advantage because it is stronger and more durable than the SSF. BWs can increase energy efficiency, but only to a certain extent
		Tools	Survey	
		Config	BH, SH, BW*	
		Mode	Natural ventilation	
	^16^	Variables	Cost, economy	Long-term savings make DSFs more affordable than SSFs, in addition to being more robust. DSFs make the environment more comfortable and environmentally friendly; they also lower maintenance costs by conserving the building's energy resources
		Tools	Review	
		Config	BH, SH, SB, BW*	
		Mode	All modes	

*BH, SH, SB, and BW stand for the building-height, storey-height, shaft-box, and box-window, respectively.

### Categorizing DSFs according to literature

DSFs are an emerging kind of building facade designed to boost the energy efficiency of glass envelopes. A DSF is described by the specific design and function of the system, which is called the configuration. In general, a DSF consists of an outer layer and an inner layer, which are separated by a cavity (air gap) of varying size. The air gap is crucial to the proper operation of both skins and providing an insulating layer air flows and exchange with the outside. DSFs have three unique layers: an inside glazed wall system, a ventilated air cavity, and an outside surface, each of which can be further broken down into four configurations: building-height, storey-height, shaft-box, and box-window DSFs^20^. Study by^31^ investigate a conceptual cavity configuration to represent the cavity's shape and volume, various key features, advantages, and disadvantages with these kind of DSFs have been further examined (Table 2).

**Table 2:** Key features of DSF typologies.

DSF	Description	Key features
Building-height (Multistorey)	The entire facade or in certain situations, a number of rooms and floors without any separators are covered by the cavity space between the inner and outer skins of the building. Vent apertures are situated close to both the roof and the ground	Throughout the height, there are no restrictions on vertical airflow
		The building’s bottom is usually where the air inlet is located
		Because the ventilation rate is not even throughout the building, this type of DSF is not suited for natural ventilation
		Concerns include noise transfer between floors and fire safety
Storey-height (Corridor)	Divided into sections at each floor level or spanning multiple floors. For each level, air vents on the external skin should be placed close to the floor and ceiling. Three different ventilation types are possible: mechanical, natural, and hybrid	There is only one floor of vertical airflow
		There is no restriction on horizontal airflow
		Each floor has air vents and inlets at the bottom and top
		Natural ventilation is made possible by this kind of DSF, which also enhances fire safety
Shaft-box	For the stack effect, this type draws air from its own cavity into unique nearby shafts that extend over a number of stories	Vertical and horizontal partitions on each floor and at each box unit limit airflow
		It is possible to achieve improved sound insulation inside the cavity and natural ventilation
Box-window	Each box has its own air circulation and is enclosed both horizontally and vertically	Combines building-high vertical shaft with storey-height cavity
		Through the inlets on each level, air enters the cavity that is one storey high and converges at the vertical shafts
		Due to buoyancy in the shaft, natural ventilation is made feasible even with minimal outside wind

**Figure 1:**
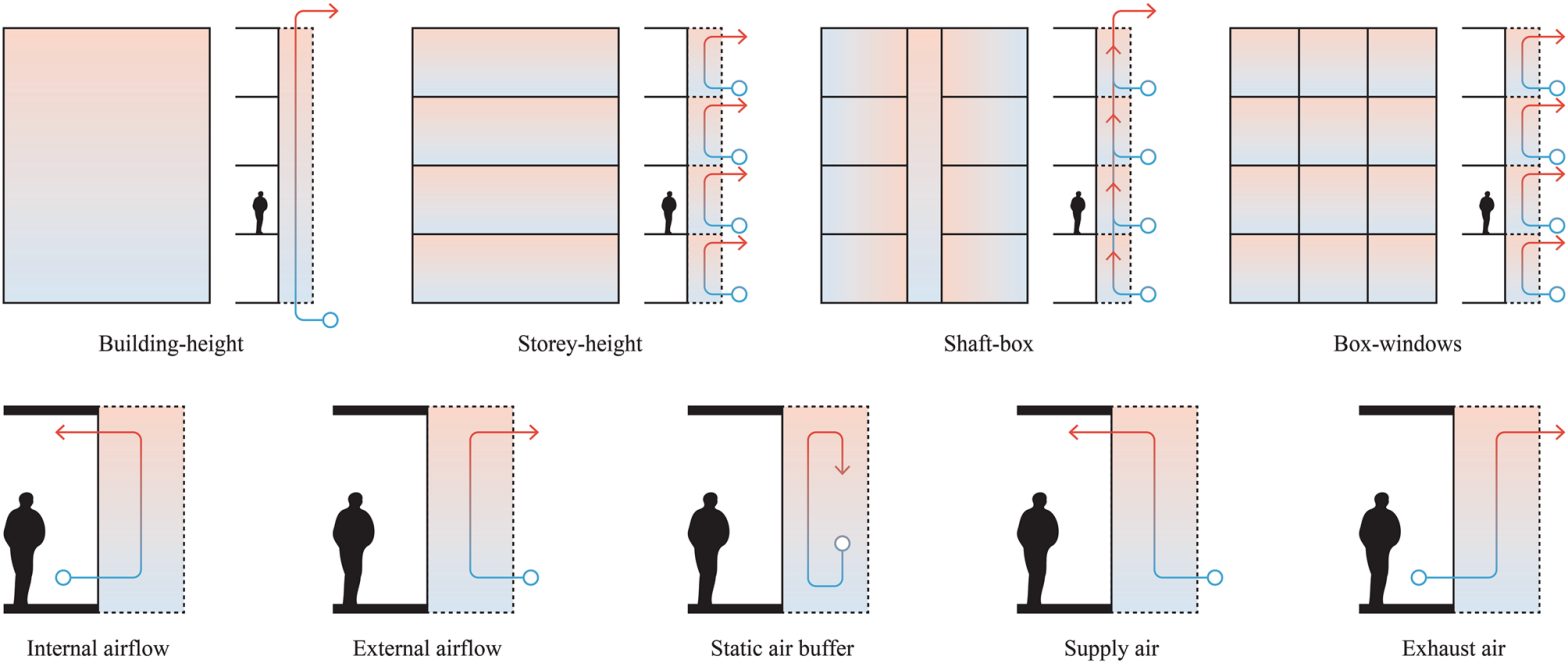
DSF configurations and ventilation modes with relation to envelope layers.

Since the initial approach can have an impact on the design stage, the classification of DSFs is crucial. It is vital to identify the design and technical parameters (such as the materials used) that can affect the function and performance of the system and the physical characteristics of the cavity after choosing a suitable type of double facade for the structure^12^. The airflow analysis within the cavity has received special attention. The airflow between two skins could be treated in several modes. In contrast to mechanical ventilation, natural ventilation is a combination of the stack effect and wind pressure in multi-storey buildings^4^. Hybrid ventilation combines mechanical and natural ventilation (Fig. 1). However, unless natural forces are insufficient to produce the desired performance, natural ventilation is exploited as much as feasible^32^.

According to the literature, DSF systems seem to be more cost-effective in the long run and more energy-efficient than SSF systems. This study examines factors like cavity width, glazing, orientation, materials, and climate zones that affect the thermal and energy performance of DSFs. Despite the potential advantages of DSFs in enhancing the energy performance of buildings, there is still a gap in real-world experience and knowledge, especially with regard to natural ventilation, geometry combinations, and construction material. The study aims to determine the best-case scenario with an appropriate double-skin facade configuration for optimizing building energy performance for each orientation while taking into consideration the hot climate in Erbil city.

## Methodology

### Overview

**Figure 2:**
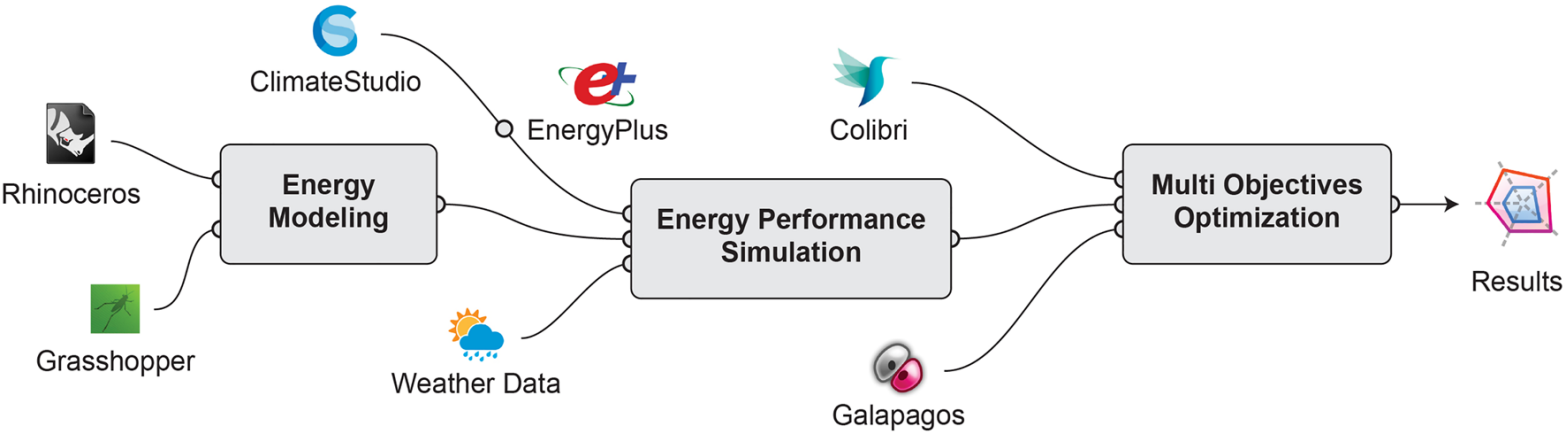
Study flowchart diagram.

Through a review of earlier research on DSFs implemented in climates similar to Erbil, this study employs a quantitative investigative approach. It also uses parameterization simulation and multi-objective optimization tools. A research framework is introduced, employing a parametric 3D energy modelling platform and a Grasshopper algorithm (Supplementary Fig. S1). In addition, an energy performance analysis of the building is modelled using the EnergyPlus simulation program that interacts with ClimateStudio (Fig. 2). The inputs where analyses by Colibri and Galapagos data solvers. The results of computer simulations are discussed and compared to better comprehend the baseline building condition relative to the improved building condition using multi-objective analysis approach.

### Energy performance metric

It is helpful to employ a normalized whole building energy consumption index, such as the energy use intensity (EUI), which is widely used for building performance analysis^33^, to incorporate the energy performance of the building into the optimization method. The building's total annual energy consumption is divided by its total gross floor area to determine the EUI^34^. This energy parameter is influenced by a wide range of internal and external elements, including weather, heating/cooling loads, and building programs. The EUI includes all the important energy usage metrics for the whole year, such as the annual heating, cooling, artificial lighting, and equipment loads. The units are expressed as kilowatt-hours used per square metre yearly (kWh/m^2^/yr).

### Optimization workflow

**Figure 3:**
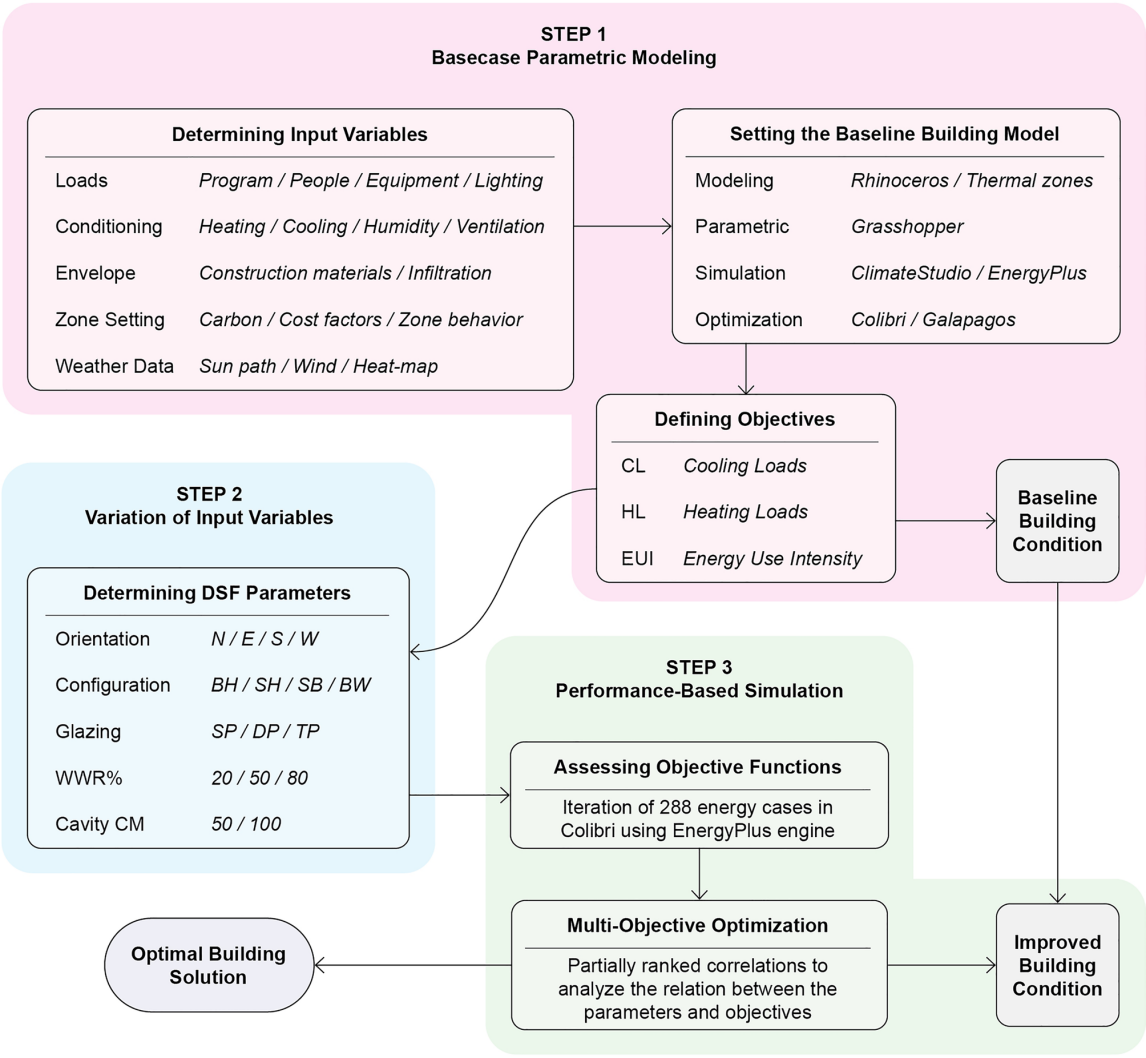
Research optimization workflow (Author).

The optimization process starts by determining input variables. The energy performance of DSF buildings depends on several factors examined by numerous studies^2,9,11,15,35^. These factors include the site, building program, orientation, heat-map, climate conditions, use of natural or mechanical ventilation between the two skins, depth of the air cavity, and material composition. Then the process led to preparing energy model as a baseline and determining thermal zones for assessing buildings initial condition regarding cooling, heating, and energy use intensity. The energy model is designed to simulate its parameters by ClimateStudio plugin, which obtains its results based on the EnergyPlus database. Variation of new input variables introduced for the model as; orientation, DSF configuration, glazing material, facade openings, and cavity depth (Supplementary Fig. S4). For assessing objective functions 288 iterations where made with Colibri and Galapagos. The width of the cavity is determined to be 50 cm and 100 cm with various WWRs of 20%, 50%, and 80%. The glazing pane varies between single, double, and triple. The walls in the building are made of concrete blocks, and low-E glass is used in the windows. The simulation results over a 1-year period according to the climate data of Erbil obtained. Multi-objective analysis is used to analyse the different parameters and determine improved building model conditions (Fig. 3).

### Setting the baseline building model

Erbil is a city in northern Iraq at 36.23°N, 43.97°E with a height 390 m above sea level that is located in the second hot ASHARE climate zone (Table 3, Supplementary Fig. S5, and Supplementary Fig. S6). Erbil is considered to have a temperate dry hot summer. In this context, the case study is evaluated in three steps (Fig. 3). First, the parameters for the area and climate of the building zone to be modelled are determined with no specific context application to generalize the results.

**Table 3:** Site analysis and weather data of the city of Erbil.

Climate zone		Heating design conditions		Cooling design conditions	
Koppen climate zone	Hot summer	Coldest month	January	Hottest month	July
ASHARE climate zone	Hot (2)	Coldest week	1/6–1/12	Hottest week	7/6–7-12
Average annual temperature	11 °C	Typical winter week	1/27–2/2	Typical summer week	8/24–8/30
Annual total solar radiation	1931 kWh/m^2^	Annual HDD for 18 °C	1040	Annual CCD for 10 °C	4329
Average annual wind speed	3 m/s, mostly east	Design temp. 0.04%	− 10 °C	Design temp. 99.6%	31.7 °C

**Figure 4:**
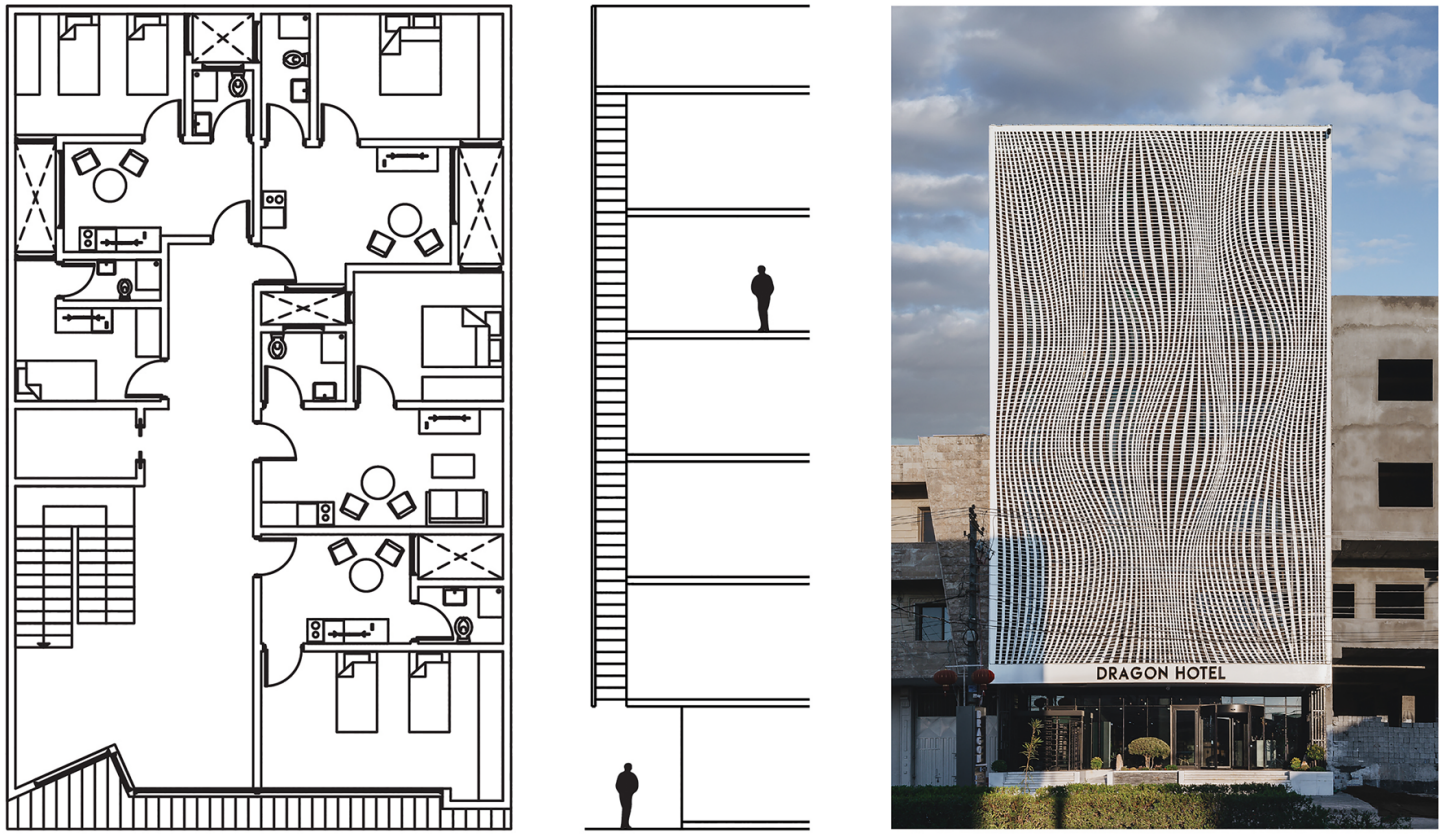
Cavity representation from layout, section, and exterior views, with permission from Sewaisi, Harem, Dragon Hotel Facade (2020).

Selection of the baseline case study is based on specified criteria: a DSF with natural ventilation cavity exposed to the outdoor climate. The floor plan layout for the baseline model is 12.5 × 20 m with 6 floors of 3.5 m height. The building consists of one facade oriented to the west and covered with a louvered building-height DSF with 50 cm cavity width, which is the naturally ventilated mode used for the cavity. Other facades are adiabatic faces and not considered within the energy performance assessment (Fig. 4).

In order to undertake a solid analysis and generalize the findings, the authors conducted a comprehensive investigation of local construction. The applied construction specifications, material characteristics, and climate data (Table 4). The main facade covered with steel panels contains air gaps that feed the cavity with natural ventilation. The application of different glazing methods is applied to optimize the initial conditions with different glass specifications. The thermal values and solar heat gain coefficient (SHGC) are measured accordingly.

**Table 4:** Construction material properties of baseline model elements.

Construction		Material layers	Thickness [m]	U-value [W/(m^2^·K)]	R-value [W/(m^2^·K)]	SHGC	Embodied energy [kWh/m^2^]	Embodied carbon [kgCO_2_/m^2^]
Building envelope	Roof	XPS boards	0.003	0.156	6.29	–	277.78	69
		Concrete	0.203					
	Facade	Stucco	0.025	3.133	0.149	–	111	54.5
		Concrete	0.203					
		Plaster	0.013					
	Partitions	Plaster	0.005	2.422	0.153	–	7.39	41.86
		Sand-lime brick	0.080					
		Plaster	0.005					
	Slab	Concrete	0.200	3.704	0.1		100	48
DSF glazing	Single pane (SP)	Sungate	0.006	3.51	–	0.744	121	34.5
	Double Low-e pane (DP)	Gray lite II	0.0057	1.26	–	0.106	119	74.735
		Krypton	0.012					
		Solarban	0.006					
	Triple Low-e pane (TP)	Clear solarban	0.0057	0.58	–	0.163	180	117
		Krypton	0.0127					
		Solarban	0.0057					
		Krypton	0.0127					
		Clear float glass	0.0058					

For more accurate energy simulation, the study calculates the median of loads used by occupancy and the usage of lights and equipment during the year. The simulation process is made more reliable by this data inquiry. The schedules for the baseline model describe the activities, occupancy, and operational schedules that will be used to employ the loads. Other specific criteria are also included, such as the room sizes, space requirements per person, relationships between areas, equipment needs, and budget (Supplementary Fig. S3).

### Setting optimized energy modelling

**Figure 5:**
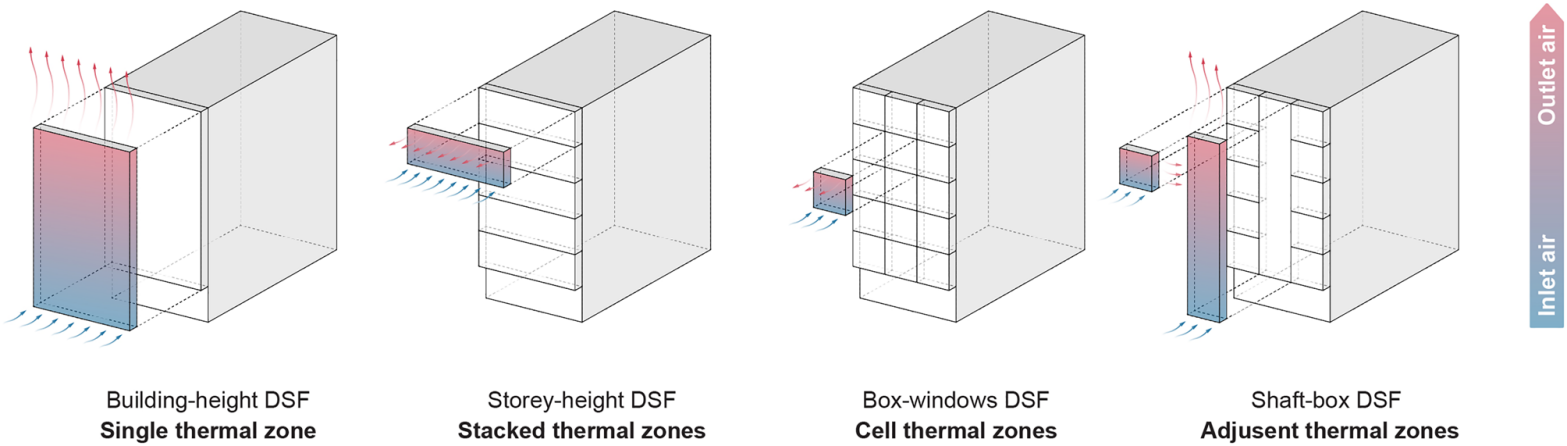
Adjacent thermal zone for the DSF cavity volume and internal spaces.

To assess the airflow inside the cavity volume, different geometrical partitions are modeled (Fig. 5). Each volume is determined by the type of DSF geometry and partitioning. With advances in literature methods, the calculation of airflow is achieved by determining the source of airflow, destination, direction, cavity depth, and geometric partitioning^12^. In this research, the adjacent thermal zone acts as an outdoor air curtain or a cavity that is predefined with the use of Grasshopper’s evolutionary algorithms. The cavity geometry was optimized, and the outcomes were generated by EnergyPlus using the ClimateStudio plugin. The simulation calculates detailed natural ventilation and mass flow through a multi-zone model with advanced airflow network (AFN) capabilities. Pressure coefficients can be auto-calculated or provided as an envelope mesh from computational fluid dynamics simulations (CFD). In all configurations, the airflow is considered to be going in the same direction, and it is assumed that enthalpy can only move in the vertical direction.

Every airflow and building component in AFN models is viewed as a network of nodes that correspond to individual rooms and subsets of rooms. For inlet and outlet airflows, the idea of mass conservation results in non-linear equations that are integrated over time to describe the flow rates. There is only one node at the centroid of each thermal zone. On the other hand, CFD simulations compute the desired flow velocity at a large number of nodes connected and distributed across the physical domain, forming what is known as a mesh or a grid. The configurations are surrounded by a two- or three-dimensional grid of nodes, and the conservation equation for mass, momentum, and thermal energy is solved for each node.

In general, the research method aims to find out best case scenario for the use different DSF configurations for each orientation using EnergyPlus and ClimateStudio. The combination between these two software will enhance the findings in the case of Erbil city.

## Results

### Baseline DSF performance

**Figure 6:**
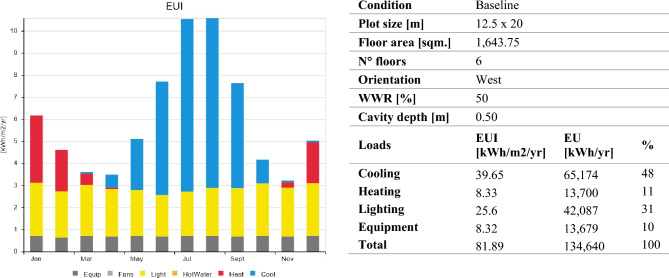
Baseline energy performance during the entire year.

According to the results obtained from the baseline model, there is significant cooling energy consumption during the hot period starting from April to October, where the building consumes 65,174 kWh, which is equal to 48% of the total load (Fig. 6). For this reason, the study concentrates on the performance of different types of DSFs by means of their energy demand for cooling loads and the effects of the applied parameters in reference to the reduction in cooling energy consumption. Lighting and equipment are not considered in this research, contributing 41% of the total energy loads. Heating energy is not used between April and October in the building; the lowest heating energy consumption is in November, and the highest consumption is in January, which does not exceed 5% of the total load. On the other hand, cooling energy is not used between November and March, while the lowest cooling energy consumption is in April, and the highest consumption is observed in July by 16,816 kWh yearly. The building's overall energy consumption is estimated to be 134,623 kWh/yr.

Hypothetical scenarios are generated for evaluating other orientations; thus, the baseline model with its properties is rotated towards other cardinal directions (north, east, and south). The cooling and heating energy loads within the entire year are determined as the initial conditions for each facade orientation. According to the data gained, the east facade receives the highest cooling loads of 43 kWh/m^2^ annually, while the lowest energy consumption occurred when the model is oriented towards the north, where the cooling rate decreases by 9%. The south facade involves the longest period of energy consumption because of the air-conditioning system and cooling energy demand from April to November, when the total cooling energy intensity exceeds 4 kWh/m^2^. The initial cooling and heating conditions regarding each facade orientation can be represented in (Table 5 and Supplementary Table S1).

**Table 5:**
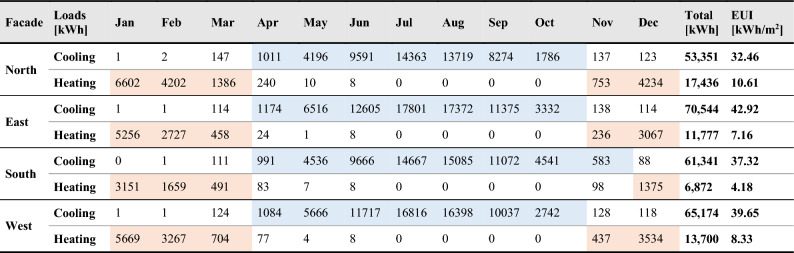
Baseline performance for monthly cooling and heating loads according to different facade orientations.

### Optimized DSF configuration performance

Scenarios with double-skin facade applied on different fronts where energy modelled using ClimateStudio. An assessment is performed according to a 1-year period in Erbil using a DSF with varying configurations on the reference baseline models with four cardinal orientations. For all oriented facades, 288 iterations for different parameters are applied using Colibri Software for data analysis. The outcomes show the consumption curves for each scenario's seasonal and annual changes (Supplementary Fig. S2).

#### North-oriented facade results

In the first scenario, the DSF is oriented in the northern direction. 72 different configurations applied. The amount of heating loads does not change as much, so the difference is 3916 kWh, which is not considered a significant improvement. However, the end-use energy savings of 6.41 kWh/m^2^ do pose a noticeable effect (Table 6). Thus, the overall curve of the northern facade in comparison to the initial building condition does not record significant improvement. The optimum solution for energy demand determined by the use of box-window configuration with triple pane DSF glazing that 80% opening. The DSF scenarios reduce the annual cooling and heating loads 9%, this saves 10,536 kWh yearly.

**Table 6:** Results of the iteration scenarios for the north facade.

Building-height DSF						Storey-height DSF						Box-window DSF						Shaft-box DSF					
Cavity depth [cm]	WWR [%]	DSF Glazing	EUI [kWh/m^2^/yr]	Cooling [kWh/m^2^/yr]	Heating [kWh/m^2^/yr]	Cavity depth [cm]	WWR [%]	DSF Glazing	EUI [kWh/m^2^/yr]	Cooling [kWh/m^2^/yr]	Heating [kWh/m^2^/yr]	Cavity depth [cm]	WWR [%]	DSF Glazing	EUI [kWh/m^2^/yr]	Cooling [kWh/m^2^/yr]	Heating [kWh/m^2^/yr]	Cavity depth [cm]	WWR [%]	DSF Glazing	EUI [kWh/m^2^/yr]	Cooling [kWh/m^2^/yr]	Heating [kWh/m^2^/yr]
50	20	SP	76.7	30.5	11.8	50	20	SP	76.8	30.7	11.7	50	20	SP	76.7	30.3	11.8	50	20	SP	76.8	30.5	11.7
100	20	SP	76.6	30.4	11.8	100	20	SP	76.9	30.7	11.7	100	20	SP	76.6	30.2	11.8	100	20	SP	76.9	30.5	11.7
50	50	SP	76.9	32.5	10.6	50	50	SP	77.1	32.6	10.6	50	50	SP	76.7	32.1	10.7	50	50	SP	76.7	32.2	10.6
100	50	SP	76.7	32.2	10.6	100	50	SP	77	32.5	10.6	100	50	SP	76.4	31.8	10.7	100	50	SP	76.5	31.9	10.7
50	80	SP	77.6	34.4	9.4	50	80	SP	77.7	34.5	9.4	50	80	SP	77.2	34.1	9.4	50	80	SP	77.2	33.9	9.5
100	80	SP	77.2	34.0	9.5	100	80	SP	77.6	34.3	9.5	100	80	SP	76.8	33.5	9.5	100	80	SP	76.8	33.4	9.6
50	20	DP	78	29.3	11.5	50	20	DP	78.1	29.5	11.5	50	20	DP	78	29.2	11.5	50	20	DP	78.1	29.4	11.4
100	20	DP	78	29.3	11.5	100	20	DP	78.2	29.6	11.4	100	20	DP	78	29.2	11.5	100	20	DP	78.1	29.5	11.3
50	50	DP	75.7	28.8	10.3	50	50	DP	75.8	28.9	10.2	50	50	DP	75.7	28.7	10.3	50	50	DP	75.8	28.8	10.2
100	50	DP	75.8	28.8	10.2	100	50	DP	75.9	29.0	10.2	100	50	DP	75.7	28.7	10.3	100	50	DP	75.9	28.9	10.2
50	80	DP	73.5	28.2	9.1	50	80	DP	73.5	28.3	9.0	50	80	DP	73.4	28.1	9.1	50	80	DP	73.5	28.2	9.0
100	80	DP	73.6	28.2	9.0	100	80	DP	73.7	28.3	9.0	100	80	DP	73.5	28.1	9.1	100	80	DP	73.7	28.2	9.0
50	20	TP	76.6	29.2	11.3	50	20	TP	76.7	29.3	11.3	50	20	TP	76.8	29.1	11.3	50	20	TP	76.9	29.3	11.2
100	20	TP	76.6	29.2	11.3	100	20	TP	76.9	29.4	11.2	100	20	TP	76.8	29.1	11.3	100	20	TP	77	29.4	11.1
50	50	TP	73.2	28.6	9.7	50	50	TP	73.3	28.7	9.7	50	50	TP	73.1	28.5	9.8	50	50	TP	73.2	28.6	9.7
100	50	TP	73.2	28.5	9.7	100	50	TP	73.4	28.7	9.7	100	50	TP	73	28.4	9.8	100	50	TP	73.2	28.6	9.7
50	80	TP	70.7	28.1	8.2	50	80	TP	70.7	28.2	8.2	50	80	TP	70.6	28.1	8.2	50	80	TP	70.6	28.1	8.2
100	80	TP	70.7	28.1	8.2	100	80	TP	70.8	28.1	8.2	100	80	TP	70.5	28.0	8.2	100	80	TP	70.6	28.0	8.2

#### East-oriented facade results

When considering DSF installation in the east-oriented facade, the simulation results are promising. All configurations show higher energy savings for a wider spacing between the envelope layers with double pane glazing at an 80% open ratio. A shaft-box DSF configurations with more cavity depth of 100 cm significantly improves natural ventilation within the air gap due to the mass of airflows in the inter-cavity space mostly observed from October to April. The cooling energy is reduced by 21,895 kWh compared to the initial condition among 61 conducted simulation scenarios. The amount of heating loads increases by 2146 kWh in comparison to the baseline. The energy usage significantly decreases by 11.4 kWh/m^2^ annually which backup 16% of energy usage yearly (Table 7).

**Table 7:** Results of the iteration scenarios for the east facade.

Building-height DSF						Storey-height DSF						Box-window DSF						Shaft-box DSF					
Cavity depth [cm]	WWR [%]	DSF Glazing	EUI [kWh/m^2^/yr]	Cooling [kWh/m^2^/yr]	Heating [kWh/m^2^/yr]	Cavity depth [cm]	WWR [%]	DSF Glazing	EUI [kWh/m^2^/yr]	Cooling [kWh/m^2^/yr]	Heating [kWh/m^2^/yr]	Cavity depth [cm]	WWR [%]	DSF Glazing	EUI [kWh/m^2^/yr]	Cooling [kWh/m^2^/yr]	Heating [kWh/m^2^/yr]	Cavity depth [cm]	WWR [%]	DSF Glazing	EUI [kWh/m^2^/yr]	Cooling [kWh/m^2^/yr]	Heating [kWh/m^2^/yr]
50	20	SP	79.7	36.5	9.4	50	20	SP	78.9	35.6	9.5	50	20	SP	79.9	36.5	9.6	50	20	SP	79	35.4	9.8
100	20	SP	79.4	36.0	9.6	100	20	SP	77.9	34.3	9.8	100	20	SP	80	36.2	9.9	100	20	SP	78.1	34.0	10.3
50	50	SP	83.8	42.9	7.2	50	50	SP	82.3	41.3	7.3	50	50	SP	84.2	43.1	7.3	50	50	SP	82.2	40.9	7.6
100	50	SP	83.2	42.0	7.5	100	50	SP	80.2	38.7	7.8	100	50	SP	84	42.5	7.8	100	50	SP	80.2	38.1	8.4
50	80	SP	89	49.8	5.5	50	80	SP	86.3	46.9	5.7	50	80	SP	89.6	50.2	5.7	50	80	SP	86.1	46.3	6.1
100	80	SP	88	48.4	5.9	100	80	SP	83.1	43.2	6.2	100	80	SP	89.1	49.2	6.2	100	80	SP	82.9	42.3	6.9
50	20	DP	77.9	32.2	10.2	50	20	DP	77.5	31.7	10.3	50	20	DP	78.2	32.4	10.3	50	20	DP	77.6	31.7	10.4
100	20	DP	77.9	32.0	10.4	100	20	DP	77.1	31.2	10.4	100	20	DP	78.2	32.3	10.4	100	20	DP	77.3	31.1	10.6
50	50	DP	75.2	31.5	9.2	50	50	DP	74.8	31.0	9.2	50	50	DP	75.4	31.6	9.2	50	50	DP	74.8	30.9	9.3
100	50	DP	75.1	31.3	9.3	100	50	DP	74.3	30.4	9.3	100	50	DP	75.4	31.5	9.4	100	50	DP	74.4	30.3	9.6
50	80	DP	73.2	30.8	8.1	50	80	DP	72.8	30.3	8.1	50	80	DP	73.4	31.0	8.1	50	80	DP	72.8	30.2	8.3
100	80	DP	73.1	30.6	8.2	100	80	DP	72.3	29.8	8.3	100	80	DP	73.4	30.8	8.3	100	80	DP	72.4	29.6	8.5
50	20	TP	76.5	32.3	10.0	50	20	TP	76	31.8	10.0	50	20	TP	76.7	32.4	10.1	50	20	TP	76.1	31.7	10.2
100	20	TP	76.4	32.1	10.1	100	20	TP	75.6	31.2	10.2	100	20	TP	76.8	32.3	10.2	100	20	TP	75.7	31.1	10.4
50	50	TP	74.4	32.1	8.5	50	50	TP	73.9	31.5	8.5	50	50	TP	74.6	32.2	8.5	50	50	TP	73.9	31.4	8.7
100	50	TP	74.3	31.8	8.6	100	50	TP	73.3	30.8	8.7	100	50	TP	74.6	32.1	8.7	100	50	TP	73.4	30.6	8.9
50	80	TP	72.7	31.9	7.0	50	80	TP	72.1	31.3	7.0	50	80	TP	72.8	32.1	7.0	50	80	TP	72.1	31.1	7.2
100	80	TP	72.5	31.6	7.1	100	80	TP	71.5	30.4	7.2	100	80	TP	72.8	31.8	7.2	100	80	TP	71.5	30.2	7.5

#### South-oriented facade results

Energy performance optimization for southern DSF facade configurations is simulated by the same iterations as the previous cases. The usage of total energy intensity decreases by 9% from the initial condition, whereas the cooling loads with this configuration also achieve good performance, decreasing from 61,341 to 46,376 kWh, showing a significant improvement. A higher cavity depth of 100 cm improves the thermal performance of indoor spaces that let fresh airs enter the cavity and helps control the amount of heat gain. This helps keep the building comfortable and reduces the need for air conditioning. The results on this orientation show that shaft-box configurations reduce the energy usage of the building from 75.36 to 69.40 kWh/m^2^. The cooling energy undergoes a notable reduction of 14,965 kWh compared to the initial condition. The amount of heating loads increases by 4104 kWh annually compared to the baseline, which is 6872 kWh. The total end-use energy savings are 5.96 kWh/m^2^ yearly (Table 8).

**Table 8:** Results of the iteration scenarios for the south facade.

Building-height DSF						Storey-height DSF						Box-window DSF						Shaft-box DSF					
Cavity depth [cm]	WWR [%]	DSF Glazing	EUI [kWh/m^2^/yr]	Cooling [kWh/m^2^/yr]	Heating [kWh/m^2^/yr]	Cavity depth [cm]	WWR [%]	DSF Glazing	EUI [kWh/m^2^/yr]	Cooling [kWh/m^2^/yr]	Heating [kWh/m^2^/yr]	Cavity depth [cm]	WWR [%]	DSF Glazing	EUI [kWh/m^2^/yr]	Cooling [kWh/m^2^/yr]	Heating [kWh/m^2^/yr]	Cavity depth [cm]	WWR [%]	DSF Glazing	EUI [kWh/m^2^/yr]	Cooling [kWh/m^2^/yr]	Heating [kWh/m^2^/yr]
50	20	SP	79.5	34.5	10.2	50	20	SP	78.2	32.8	10.6	50	20	SP	80	34.6	10.4	50	20	SP	79.3	33.8	10.5
100	20	SP	79.3	34.2	10.4	100	20	SP	80.7	38.3	8.5	100	20	SP	80.1	34.4	10.6	100	20	SP	78.8	32.7	10.9
50	50	SP	81.9	39.6	8.3	50	50	SP	79.1	36.1	9.0	50	50	SP	82.2	39.8	8.5	50	50	SP	80.7	38.0	8.8
100	50	SP	81.5	38.9	8.6	100	50	SP	83.4	42.6	7.0	100	50	SP	82.2	39.4	8.9	100	50	SP	79.2	35.8	9.4
50	80	SP	85.6	45.1	6.8	50	80	SP	81	39.6	7.5	50	80	SP	86.1	45.4	6.9	50	80	SP	83.3	42.2	7.3
100	80	SP	84.8	43.9	7.2	100	80	SP	79	31.0	10.7	100	80	SP	85.8	44.6	7.4	100	80	SP	80.9	38.9	8.1
50	20	DP	79.3	31.3	10.7	50	20	DP	78.7	30.6	10.8	50	20	DP	79.5	31.4	10.7	50	20	DP	79.1	31.0	10.8
100	20	DP	79.3	31.2	10.8	100	20	DP	76.9	30.4	9.6	100	20	DP	79.5	31.4	10.8	100	20	DP	78.8	30.6	10.9
50	50	DP	77.3	30.8	9.6	50	50	DP	76.7	30.0	9.7	50	50	DP	77.4	30.9	9.6	50	50	DP	77	30.4	9.7
100	50	DP	77.2	30.6	9.6	100	50	DP	75	29.8	8.5	100	50	DP	77.4	30.8	9.7	100	50	DP	76.7	30.0	9.8
50	80	DP	75.3	30.2	8.5	50	80	DP	74.7	29.4	8.6	50	80	DP	75.4	30.3	8.5	50	80	DP	75	29.8	8.6
100	80	DP	75.2	30.0	8.5	100	80	DP	78	31.0	10.5	100	80	DP	75.4	30.2	8.6	100	80	DP	74.7	29.3	8.7
50	20	TP	78.3	31.4	10.4	50	20	TP	77.7	30.6	10.6	50	20	TP	78.7	31.5	10.5	50	20	TP	78.3	31.0	10.6
100	20	TP	78.3	31.3	10.5	100	20	TP	74.8	30.6	8.9	100	20	TP	78.7	31.5	10.6	100	20	TP	78	30.6	10.7
50	50	TP	75.2	31.1	8.9	50	50	TP	74.5	30.1	9.1	50	50	TP	75.3	31.2	9.0	50	50	TP	74.9	30.6	9.1
100	50	TP	75.2	30.9	9.0	100	50	TP	72.4	30.3	7.5	100	50	TP	75.4	31.0	9.1	100	50	TP	74.6	30.0	9.2
50	80	TP	72.9	30.8	7.4	50	80	TP	72	29.6	7.6	50	80	TP	73	30.9	7.5	50	80	TP	72.4	30.2	7.6
100	80	TP	72.7	30.6	7.5	100	80	TP	80	34.6	10.4	100	80	TP	73	30.8	7.6	100	80	TP	72	29.5	7.8

#### West-oriented facade

The assessment of the west facade performed over a 1-year period in Erbil. The results provide annual consumption curves of each scenario where the amount of energy consumption is reduced among 56 scenarios (Table 9). The total energy use intensity decreases by 7.18 kWh/m^2^ in comparison to the initial condition. The cooling loads with this configuration also achieve good performance, decreasing the load from 65,174 to 48,172 kWh. The thermal performance of indoor spaces is greatly improved by application of double pane glazing system for covering DSF surface and providing 100 cm cavity depth for the cavity, which helps decrease heat loss in the winter and gain in the summer. This increases the building's energy efficiency. Compared to the initial condition, the cooling energy is reduced by 17,002 kWh, while the yearly heating loads increase by 579 kWh. The end-use energy usage significantly decreases by 7.18 kWh/m^2^ annually.

**Table 9:** Results of the iteration scenarios for the west facade.

Building-height DSF						Storey-height DSF						Box-window DSF						Shaft-box DSF					
Cavity depth [cm]	WWR [%]	DSF Glazing	EUI [kWh/m^2^/yr]	Cooling [kWh/m^2^/yr]	Heating [kWh/m^2^/yr]	Cavity depth [cm]	WWR [%]	DSF Glazing	EUI [kWh/m^2^/yr]	Cooling [kWh/m^2^/yr]	Heating [kWh/m^2^/yr]	Cavity depth [cm]	WWR [%]	DSF Glazing	EUI [kWh/m^2^/yr]	Cooling [kWh/m^2^/yr]	Heating [kWh/m^2^/yr]	Cavity depth [cm]	WWR [%]	DSF Glazing	EUI [kWh/m^2^/yr]	Cooling [kWh/m^2^/yr]	Heating [kWh/m^2^/yr]
50	20	SP	79.7	36.5	9.4	50	20	SP	78.9	35.6	9.5	50	20	SP	79.9	36.5	9.6	50	20	SP	79	35.4	9.8
100	20	SP	79.4	36.0	9.6	100	20	SP	77.9	34.3	9.8	100	20	SP	80	36.2	9.9	100	20	SP	78.1	34.0	10.3
50	50	SP	83.8	42.9	7.2	50	50	SP	82.3	41.3	7.3	50	50	SP	84.2	43.1	7.3	50	50	SP	82.2	40.9	7.6
100	50	SP	83.2	42.0	7.5	100	50	SP	80.2	38.7	7.8	100	50	SP	84	42.5	7.8	100	50	SP	80.2	38.1	8.4
50	80	SP	89	49.8	5.5	50	80	SP	86.3	46.9	5.7	50	80	SP	89.6	50.2	5.7	50	80	SP	86.1	46.3	6.1
100	80	SP	88	48.4	5.9	100	80	SP	83.1	43.2	6.2	100	80	SP	89.1	49.2	6.2	100	80	SP	82.9	42.3	6.9
50	20	DP	77.9	32.2	10.2	50	20	DP	77.5	31.7	10.3	50	20	DP	78.2	32.4	10.3	50	20	DP	77.6	31.7	10.4
100	20	DP	77.9	32.0	10.4	100	20	DP	77.1	31.2	10.4	100	20	DP	78.2	32.3	10.4	100	20	DP	77.3	31.1	10.6
50	50	DP	75.2	31.5	9.2	50	50	DP	74.8	31.0	9.2	50	50	DP	75.4	31.6	9.2	50	50	DP	74.8	30.9	9.3
100	50	DP	75.1	31.3	9.3	100	50	DP	74.3	30.4	9.3	100	50	DP	75.4	31.5	9.4	100	50	DP	74.4	30.3	9.6
50	80	DP	73.2	30.8	8.1	50	80	DP	72.8	30.3	8.1	50	80	DP	73.4	31.0	8.1	50	80	DP	72.8	30.2	8.3
100	80	DP	73.1	30.6	8.2	100	80	DP	72.3	29.8	8.3	100	80	DP	73.4	30.8	8.3	100	80	DP	72.4	29.6	8.5
50	20	TP	76.5	32.3	10.0	50	20	TP	76	31.8	10.0	50	20	TP	76.7	32.4	10.1	50	20	TP	76.1	31.7	10.2
100	20	TP	76.4	32.1	10.1	100	20	TP	75.6	31.2	10.2	100	20	TP	76.8	32.3	10.2	100	20	TP	75.7	31.1	10.4
50	50	TP	74.4	32.1	8.5	50	50	TP	73.9	31.5	8.5	50	50	TP	74.6	32.2	8.5	50	50	TP	73.9	31.4	8.7
100	50	TP	74.3	31.8	8.6	100	50	TP	73.3	30.8	8.7	100	50	TP	74.6	32.1	8.7	100	50	TP	73.4	30.6	8.9
50	80	TP	72.7	31.9	7.0	50	80	TP	72.1	31.3	7.0	50	80	TP	72.8	32.1	7.0	50	80	TP	72.1	31.1	7.2
100	80	TP	72.5	31.6	7.1	100	80	TP	71.5	30.4	7.2	100	80	TP	72.8	31.8	7.2	100	80	TP	71.5	30.2	7.5

### Analysis of design variants

Following the above-mentioned optimization dataset that analysed with Colibri software, optimal solution with the updated input sliders is chosen in parametric design software to identify high-performance structural solutions. Multi-objective optimization (MOO) is used, providing a synthetic perspective of all findings, to further study the influence of the various parameters that define each scenario as well as the relationship between those parameters and the performance indicators. An MOO holding outcomes for all DSF configurations and scenarios is produced using the interactive Design Explorer web tool^36^, as demonstrated in.

**Figure 7:**
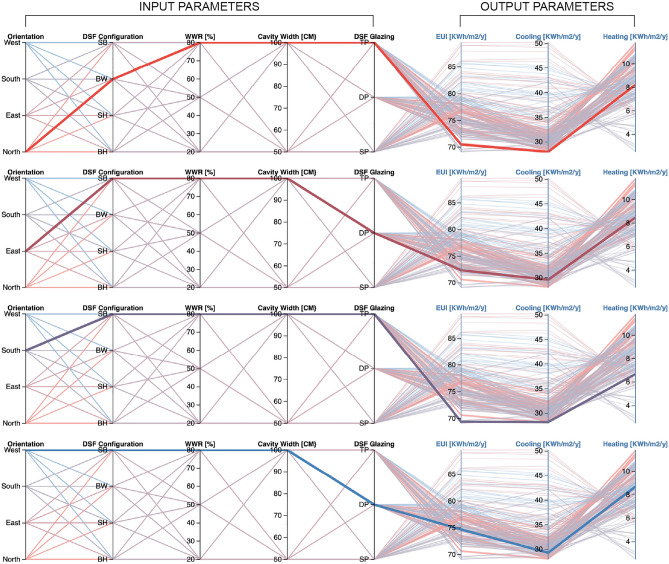
Multi-objective optimization analysis for defining the best-case scenario for each orientation.

Each evaluated scenario is represented by a line linking various vertical axes on the MOO. Each vertical axis corresponds to an input parameter that defines the scenario or an output parameter with assessed indicators such as the EUI, cooling, and heating use. By choosing values for one or more of the axes of the EUI indicator, the web tool allows chart interactivity. The MOO partially ranked correlations to analyse the relation between the parameters and objectives. The output graph displays only the scenarios that fall inside the highlighted range and excludes the rest. Only possibilities (Box-windows-shaft-box; see DSF Configuration axis) are visible for an EUI of at least 25% or lower, indicating that only those options satisfy the optimal condition (Fig. 7).

Finally, a sample of optimized DSF configuration is presented to illustrate the thermal performance of the optimally selected scenarios. A balance is reached between enhancing energy performance, lowering cooling loads, and decreasing end-use energy consumption (Table 10 and Supplementary Table S2).

**Table 10:**
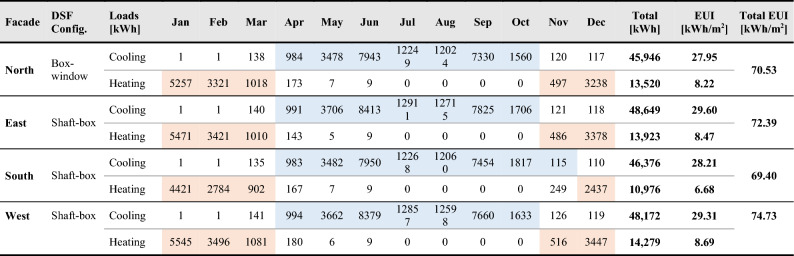
Optimized DSF performance for monthly cooling and heating loads according to different facade orientations.

## Discussion

**Figure 8:**
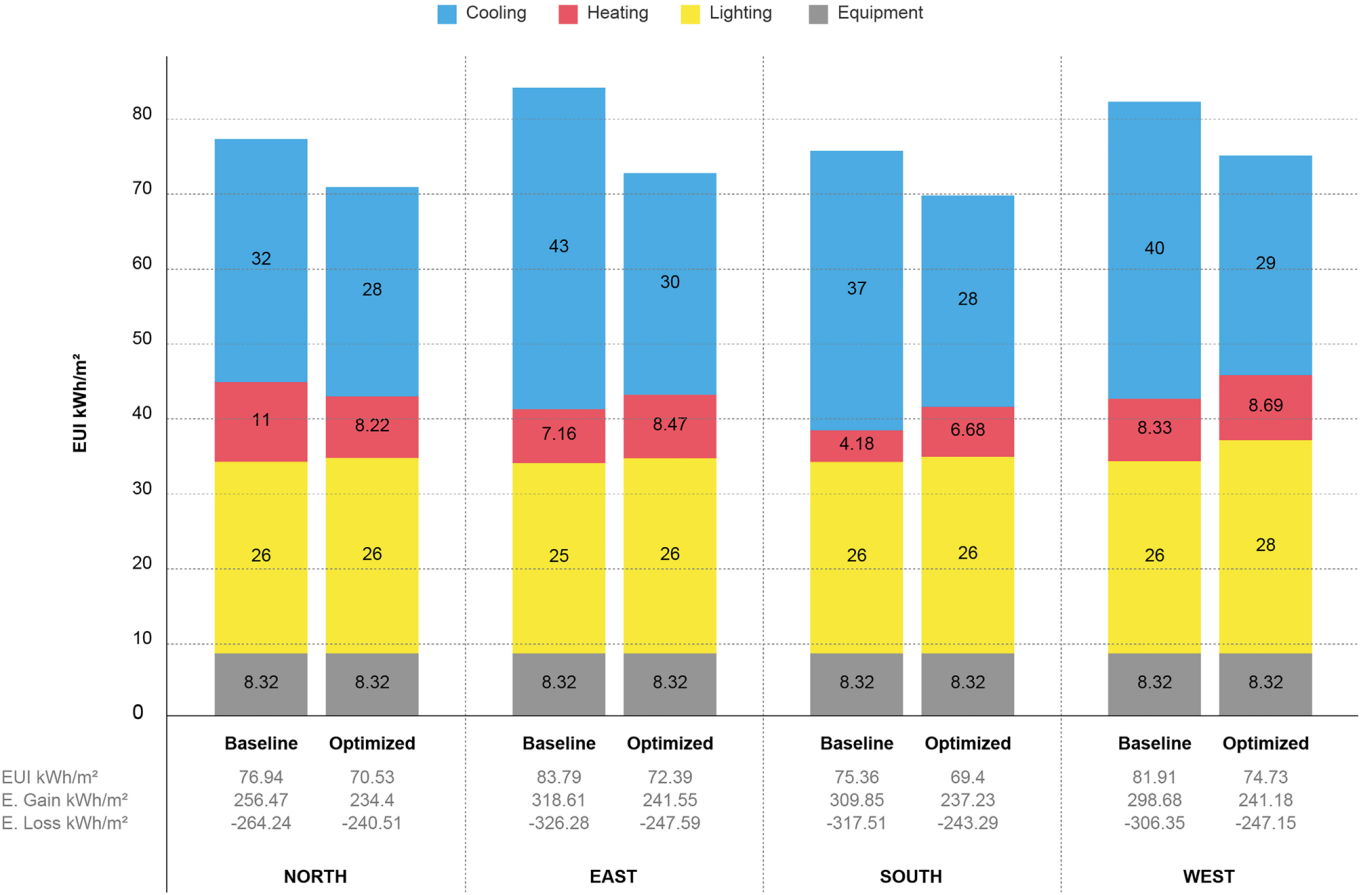
Comparison between baseline and optimized model performance.

The annual energy use comparison for optimized DSF configuration with the baseline building condition is presented. The results indicates that the proposed configuration models save energy compared with the baseline model. The study demonstrate a correlation between various DSF types on thermal performance is varies little, as well examined by^20,23^. On average, the initial energy use intensity (EUI) for baseline condition was 81.91 kWh/m^2^/yr. This translates to EUI of 74.73 kWh/m^2^/yr. The end-use energy savings ranges from 8 to 10 kWh per square meter yearly. Figure 8 summarizes the strategies used to reduce cooling consumption. The initial condition of the building and the optimized condition were compared. See also Supplementary Table S3 for a concise overview of the assessment process.

Among all iterations, energy performance achieved with multi-layering glazing parameters, this indicate that double-pane and triple-pane glass are the most appropriate for achieving an optimal solution. The majority of cases demonstrate optimum energy usage with a 100 cm cavity depth and triple glazing with 80% WWR. The analysis supports the theory that for all opaque materials, EUI decreased when WWR increased, and increased when the cavity width increased^25,26^.

During the summer, the cooling loads do not exceed 13,000 kWh, where the optimization rates between 18 and 26% compared to the building initial cooling condition. Additionally, less energy is needed because of the cavity depth protection used in the DSFs on the east, south, and west. This is because a building can lose the additional energy it gains from the north side during the summer. Similar to^9^, The study conduct that each orientation requires a different type of DSF selection. However, appropriate DSF require precise location data to determining the configuration that fit to its context. For the case of Erbil city building-height DSF acts as the best retrofit when the building facing north, while shaft-box provides the best outputs for other orientations. This is due to the naturally ventilation airflow that accelerate heat change inside cavity volume^24^. Thus, clarify that the DSF has the ability to reduce energy consumption in hot temperature conditions when the designs operate with greater airflow within the cavity space.

The optimized configuration for north orientation is box-window DSF with a 100 cm cavity width and triple pane glazing material of 80% opening provide the best retrofit. The maximum wall thermal resistance was found in box-window facade reducing the cooling energy by 7405 kWh compared to the initial condition regarding north facade application. This is verified among 61 scenarios carried out using multi-objective analysis. For east, shaft-box configurations affect the reduction in end-use energy of the building by a range of 14% with a year from 83.79 to 72.39 kWh/m^2^. The MOO for south indicates that shaft-box configuration show better performance with wider cavity depth and the use of triple pane glazing. Also, for west the shaft-box DSF with a 100 cm cavity width and triple pane of 80% opening is the best retrofit, reducing the cooling energy use of the building by 12% from the initial condition, while the annual cooling and heating energy consumption of the optimized model are found to be 29.62 kWh/m^2^ and 7.62 kWh/m^2^ annually.

The results show that the DSF system may improve energy efficiency by 9% to 14% when compared to the case study’s initial condition.

## Conclusion

A building’s facade can possibly save much energy if it is thoughtfully designed. One of the most effective ways to protect internal environments from the effects of climate change and other environmental hazards is to use DSFs. This research examined how various DSF configurations can help buildings in Erbil’s hot climate perform better in terms of energy efficiency. To that end, various scenarios were applied to DSF-covered current projects. The cavity size, WWR, and material usage comprised the DSF simulation model parameters. EnergyPlus and ClimateStudio software were used to simulate 288 distinct scenarios to track the annual energy use during the heating and cooling seasons in accordance with weather data for Erbil.

An annual cooling demand reduction of 9% to 14% was observed when a DSF configuration was incorporated into building design, demonstrating considerable benefits in energy performance. The energy effectiveness of various DSF configurations varies, with the shaft-box and box-window configurations being the most effective, followed by the 100 cm cavity depth that is naturally ventilated and wider, which exhibited an improvement in energy performance of up to 6% when compared to a cavity depth of 50 cm and a reduction in cooling energy consumption of 16% when compared to the baseline building model. The maximum wall thermal resistance was found in box-window facades. Utilizing DSFs can result in annual energy savings of up to 116,574 kWh in hot regions such as Erbil. The performance of the DSFs is impacted by their orientation. The east, south, and west facades, which perform the worst in a hot climate, are improved by the optimized DSF in all case situations by cooling loads of between 18 and 26%. These results should be taken into account when considering how selecting appropriate DSF type that match building condition.

The contribution of this study can be formulated as follows: In the case of the hot climate of Erbil, DSFs achieve potential improvements for saving energy. For north-facing facades, box-window configuration indicates the optimal solution. However, no significant improvements are noticed, indicating that the suggested systems may not be applicable for north facades. The east, south, and west shaft-box DSFs are more promising. A wider cavity space effects the distribution of airflow through the inner pane, so naturally occurring ventilation is very important. The analysis could serve as a guide for selecting the proper DSF configuration according to the desired orientation. Based on what was mentioned, the study recommends using shaft-box and box-window DSF configurations as a technically feasible and appropriate energy-saving solution, especially in the summer instances in the case of Erbil city. Thus, it is profitable to apply this system to some parts of the new facilities and to others for the new designs if the government or the investors can take the financial feasibility of this energy-saving strategy into account in their plans. As a result, the electricity demand in the summer scenario decreased to some extent.

DSF systems should be looked at more closely so that a better balance can be found between the extra cost of an extra building skin and the long-term savings that can be made by using less energy each year. Even using simulation tools, it is difficult to calculate DSF’s attitude accurately without committing certain errors. The results of EnergyPlus and ClimateStudio can only be taken as estimates without the physical experimentation that is going to be part of future research. Accordingly, the authors of this study recommend further research and development in the fields of DSF design, environmental impact, human psychology and ergonomics, and comfort for the city of Erbil. Since DSF applications in buildings are both cost-effective and energy-efficient, they should be actively pursued as a part of the solution to the issues posed by climate change and environmental hazards.

## Supplementary Information

Supplementary Information 1.

Supplementary Information 2.

Supplementary Information 3.

Supplementary Information 4.

Supplementary Information 5.

## Data Availability

The data underlying the results presented in the study are included within the manuscript.

## Abbreviations

AFN: Airflow network
BES: Building energy science
CFD: Computational fluid dynamics
DSF: Double-skin facade
EUI: Energy use intensity
IES: Integrated environmental solution
LCA: Life-cycle assessment
MOO: Multi-objective optimization
SSF: Single-skin facade
SHGC: Solar heat gain coefficient
TAS: Thermal analysis software
WWR: Window-to-wall ratio
