# Supplementary material for: The role of double-skin facade configurations in optimizing building energy performance in Erbil city
Source: Sci Rep. 2023 May 24;13:8394. doi: 10.1038/s41598-023-35555-0 (PMC10209076; doi:10.1038/s41598-023-35555-0)
Supplement: Supplementary file 1 — Supplementary Information 1. [file 41598_2023_35555_MOESM1_ESM.docx]

Supplementary Information Guide for:

The Role of Double-Skin Facade Configurations in Optimizing Building Energy Performance in Erbil City

**Mohammed Siyamand Naddaf ^1,*^, Salahaddin Yasin Baper ^1^**

^1^ Architectural Engineering Department, Salahaddin University, Erbil 44001, Iraq; salahaddin.baper@su.edu.krd

^*^ Correspondence: mohammed.taher@su.edu.krd

**Note:** This guide contains title and text summary for these categories**:**

- Supplementary Figures (S1 to S6).
- Supplementary Tables (Table S1, Table S2, and Table S3).
- Supplementary Data (File descriptions)

*The content of supplementary figures and tables presented in a single PDF file titled “Supplementary Information” out of this guide.

# Supplementary Figures:

Supplementary Fig. S1

Grasshopper definition for baseline model simulation.

Supplementary Fig. S2

Grasshopper definition for optimized model simulation.

Supplementary Fig. S3

Thermal zone schedule settings according to selected case study.

Supplementary Fig. S4

Research variables, input and output parameters.

Supplementary Fig. S5

Erbil AP Diurnal Averages.

Supplementary Fig. S6

Erbil AP Psychrometric Chart.

# Supplementary Tables:

Supplementary Table S1

Baseline building condition and energy performance results for entire year.

Supplementary Table S2

Optimized building condition and energy performance results for entire year.

Supplementary Table S3

ClimateStudio outputs, a concise overview of the assessment process involving the utilization of double-skin facade systems. It includes a comparative analysis between a baseline model and an optimized model, highlighting their respective performance outputs with a focus on thermal energy insights.

# Supplementary Data:

**Supplementary Data 1 - Rhino3D for case study model.3dm**

This file provides a 3D model for the baseline model, integrated with a Grasshopper definition to facilitate the optimization process and explore various geometric configurations of double-skin facade systems.

**Supplementary Data 2 - Grasshopper definition.gh**

This Grasshopper definition serves as a computational framework that enables the optimization process and facilitates the exploration of different geometric forms for double-skin facade configurations. By utilizing Grasshopper's visual interface, users can define and adjust various parameters and design constraints, allowing for efficient iteration and analysis of multiple design options.

**Supplementary Data 3 - Illustration of diagrams.eps**

The accompanying manuscript includes a collection of essential drawing and diagram files in their raw format. These files serve as valuable visual aids, offering comprehensive illustrations to enhance the understanding and presentation of the research findings.

# Note

The software used for simulation and visuals in this manuscript are:

- ClimateStudio v1.8
- Rhinoceros v7
- Grasshopper v7
- SketchUp Pro v2022
- Adobe Illustrator v2020
