# Supplementary material for: The role of double-skin facade configurations in optimizing building energy performance in Erbil city
Source: Sci Rep. 2023 May 24;13:8394. doi: 10.1038/s41598-023-35555-0 (PMC10209076; doi:10.1038/s41598-023-35555-0)
Supplement: Supplementary file 5 — Supplementary Information 5. [file 41598_2023_35555_MOESM5_ESM.pdf]

Supplementary Information for:

The Role of Double-Skin Facade Configurations in Optimizing Building Energy Performance in Erbil City

Mohammed Siyamand Naddaf [1,*], Salahaddin Yasin Baper [1]

[1] Architectural Engineering Department, Salahaddin University, Erbil 44001, Iraq; salahaddin.baper@su.edu.krd

[*] Correspondence: mohammed.taher@su.edu.krd

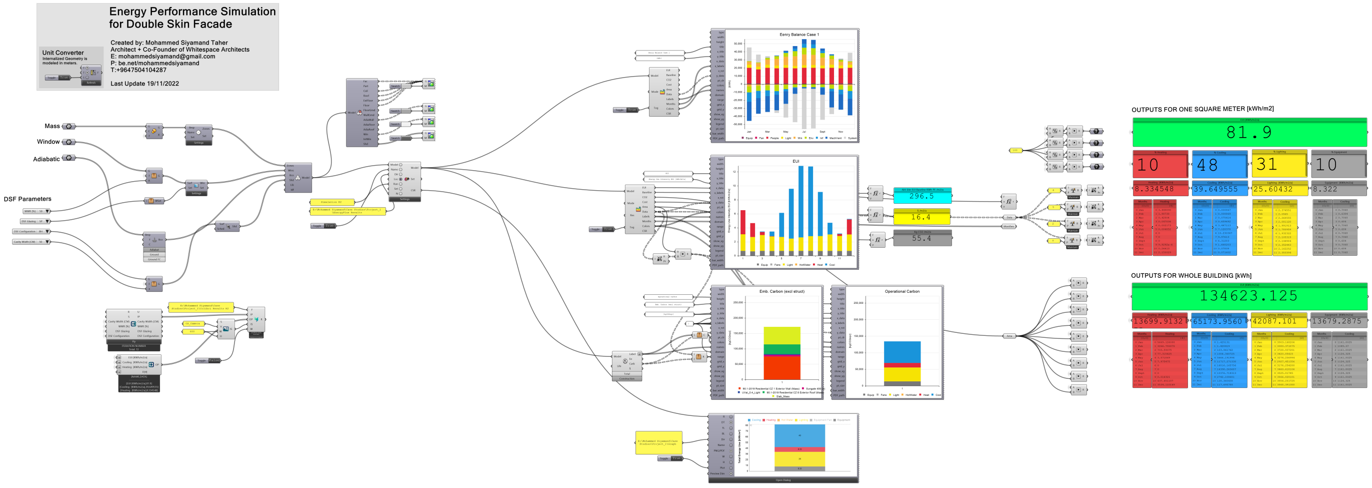

Supplementary Fig. S1 Grasshopper definition for baseline model simulation.

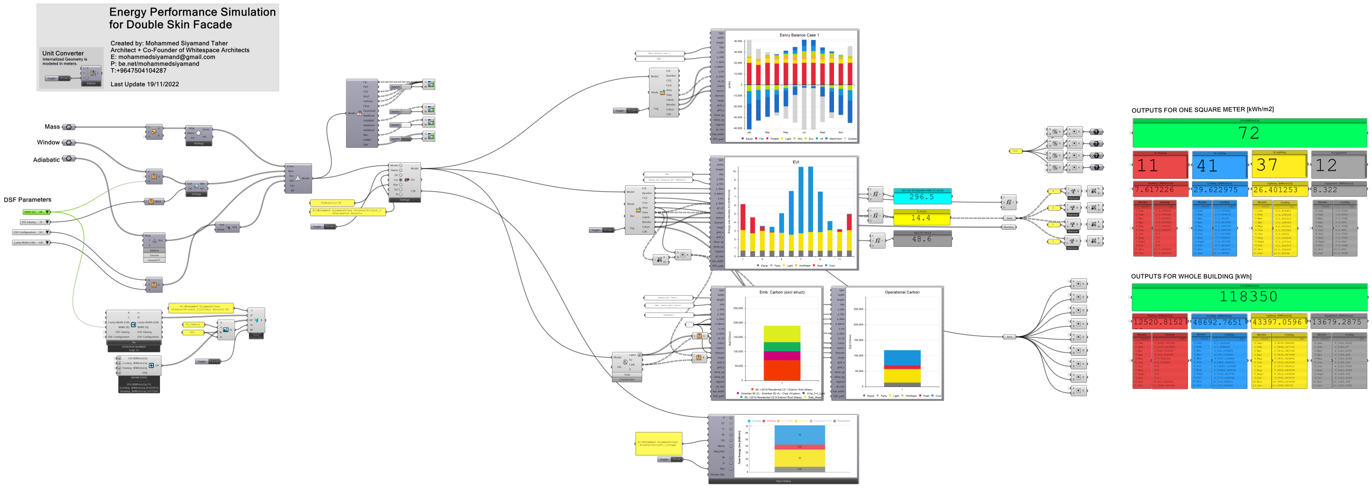

Supplementary Fig. S2 Grasshopper CS simulation findings for optimized model simulation.

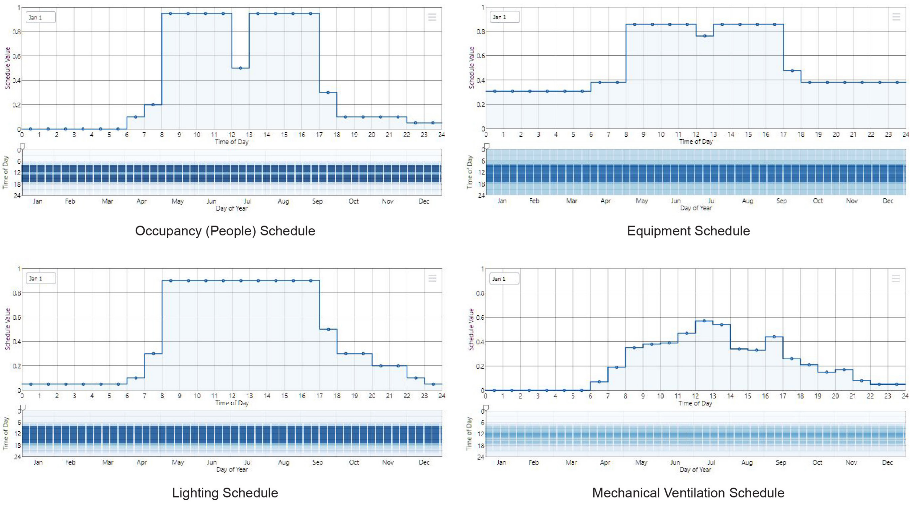

Supplementary Fig. S3 Thermal zone schedule settings.

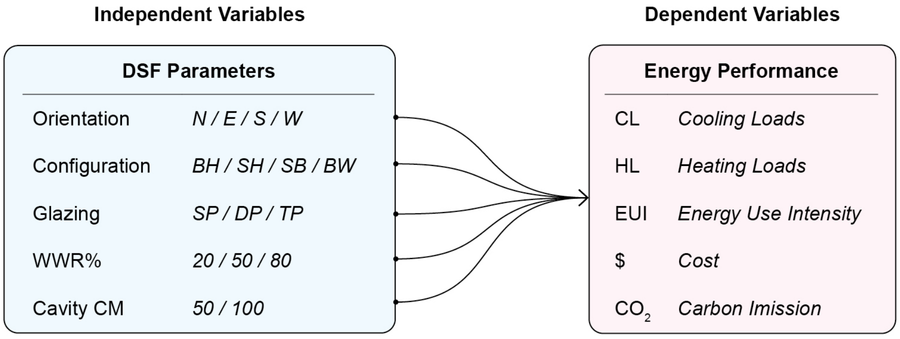

Supplementary Fig. S4 Research variables, input and output parameters.

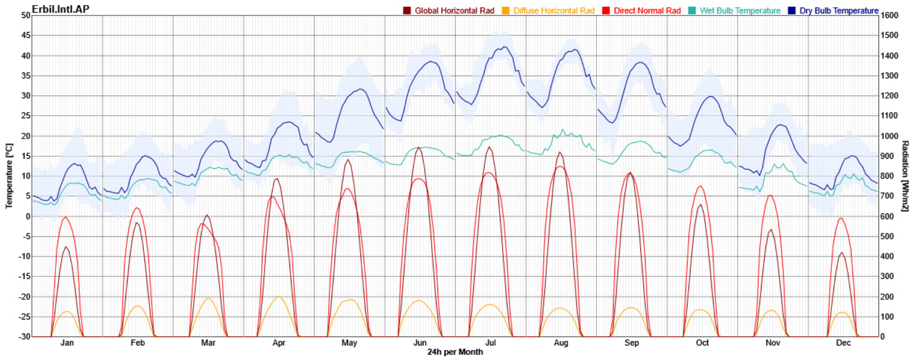

Supplementary Fig. S5 Erbil AP Diurnal Averages.

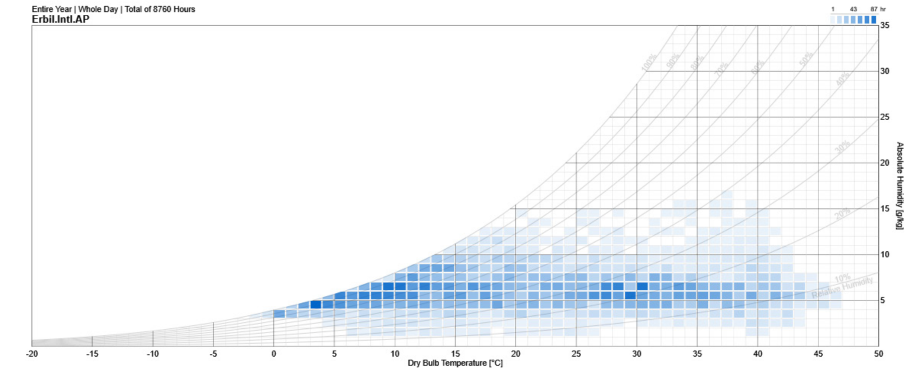

Supplementary Fig. S6 Erbil AP Psychrometric Chart.

	North		East		South		West	
	**Cooling**	**Heating**	**Cooling**	**Heating**	**Cooling**	**Heating**	**Cooling**	**Heating**
Jan	1	6602	1	5256	0	3151	1	5669
Feb	2	4202	1	2727	1	1659	1	3267
Mar	147	1386	114	458	111	491	124	704
Apr	1011	240	1174	24	991	83	1084	77
May	4196	10	6516	1	4536	7	5666	4
Jun	9591	8	12605	8	9666	8	11717	8
Jul	14363	0	17801	0	14667	0	16816	0
Aug	13719	0	17372	0	15085	0	16398	0
Sep	8274	0	11375	0	11072	0	10377	0
Oct	1786	0	3332	0	4541	0	2742	0
Nov	137	753	138	236	583	98	128	437
Des	123	4234	114	3067	88	1375	118	3534
**kWh**	**53351**	**17436**	**70544**	**11777**	**61341**	**6872**	**65174**	**13700**

Supplementary Table S1 Baseline building condition and energy performance results for entire year.

	North		East		South		West	
	**Cooling**	**Heating**	**Cooling**	**Heating**	**Cooling**	**Heating**	**Cooling**	**Heating**
Jan	1	5257	1	5471	1	4421	1	5545
Feb	1	3321	1	3421	1	2784	1	3496
Mar	138	1018	140	1010	135	902	141	1081
Apr	984	173	991	143	983	167	994	180
May	3478	7	3706	5	3482	7	3662	6
Jun	7943	9	8413	9	7950	9	8379	9
Jul	12249	0	12911	0	12268	0	12857	0
Aug	12024	0	12715	0	12060	0	12598	0
Sep	7330	0	7825	0	7454	0	7660	0
Oct	1560	0	1706	0	1817	0	1633	0
Nov	120	497	121	486	115	249	126	516
Des	117	3238	118	3378	110	2437	119	3447
**kWh**	**45946**	**13520**	**48649**	**13923**	**46376**	**10976**	**48172**	**14279**

Supplementary Table S2 Optimized building condition and energy performance results for entire year.

	lorth Simulation R	Opt.North	East Simulation R3	Opt.East	outh Simulation R	Opt.South	Vest Simulation R3	Opt.West
Total Energy Use [kWh/m²]	76.94	70.53	83.79	72.39	75.36	69.4	81.91	74.73
Cooling [kWh/m²]	32.46	27.95	42.92	29.6	37.32	28.21	39.65	29.31
Heating [kWh/m²]	10.61	8.22	7.16	8.47	4.18	6.68	8.33	8.69
Hot Water [kWh/m²]	0	0	0	0	0	0	0	0
Lighting [kWh/m²]	25.55	26.03	25.38	26	25.54	26.19	25.6	28.41
Equipment Fan [kWh/m²]	0	0	0	0	0	0	0	0
Equipment [kWh/m²]	8.32	8.32	8.32	8.32	8.32	8.32	8.32	8.32
Total Energy Use [kWh]	126462.84	115935.59	137723.72	118988.8	123879.14	114073.43	134640.26	122829.67
Cooling [kWh]	53350.79	45945.79	70543.72	48648.94	61341.36	46376.28	65173.96	48172.03
Heating [kWh]	17435.91	13519.76	11776.67	13923.07	6872.45	10975.87	13699.91	14279.06
Hot Water [kWh]	0	0	0	0	0	0	0	0
Lighting [kWh]	41996.86	42790.75	41724.04	42737.5	41986.04	43042	42087.1	46699.3
Equipment Fan [kWh]	0	0	0	0	0	0	0	0
Equipment [kWh]	13679.29	13679.29	13679.29	13679.29	13679.29	13679.29	13679.29	13679.29
Operational Energy Co2 [KgCO2/yr]	85488.88	78372.46	93101.24	80436.43	83742.3	77113.64	91016.81	83032.86
Operational Energy Co2 [KgCO2/yr/m²]	52.01	47.68	56.64	48.93	50.95	46.91	55.37	50.51
Operational Energy Cost [$/yr]	25292.57	23187.12	27544.74	23797.76	24775.83	22814.69	26928.05	24565.93
Operational Energy Cost [$/yr/m²]	15.39	14.11	16.76	14.48	15.07	13.88	16.38	14.95
Energy Gain [kWh]	421572.85	385296.49	523716.62	397050.86	509322.26	389948.83	490954.16	396433.75
Energy Loss [kWh]	-434336.79	-395344.21	-536329.9	-406980.53	-521899.92	-399910.59	-503565.53	-406250.31
System (Gain) [kWh]	52307.73	40559.27	35330.01	41769.22	20617.34	32927.6	41099.74	42837.17
System (Loss) [kWh]	-160052.36	-137837.38	-211631.17	-145946.82	-184024.09	-139128.84	-195521.87	-144516.1
Mech. Vent (Gain) [kWh]	20894.37	20943.55	20782.64	20904.26	21013.31	20983.67	20755.49	20930.53
Mech. Vent (Loss) [kWh]	-163778.09	-164733.21	-182069.47	-165412.9	-195057.24	-168510.29	-175404.34	-164794.94
Inf (Loss) [kWh]	-54659.17	-54724.38	-56509.07	-54817.68	-57555.68	-55114.04	-55784.69	-54737.55
Inf (Gain) [kWh]	17948.74	17926.91	17903.38	17929.94	17874.59	17898.6	17898.6	17930.67
Env (Gain) [kWh]	22609.98	24664.82	61220	26564.15	58365.81	25951.34	48082.02	26266.67
Env (Loss) [kWh]	-42759.38	-35877.3	-72784	-34318.56	-71601.29	-35141.58	-63471.47	-35208.02
Window (Gain) [kWh]	40322.92	12918.93	121264.3	21653.53	123972.92	23653.37	95538.96	16277.17
Window (Loss) [kWh]	-13087.79	-2171.94	-13336.18	-6484.57	-13661.63	-2015.85	-13383.16	-6993.7
Fan (Gain) [kWh]	0	0	0	0	0	0	0	0
Lighting (Gain) [kWh]	41996.86	42790.75	41724.04	42737.5	41986.04	43042	42087.1	46699.3
People (Gain) [kWh]	225492.25	225492.25	225492.25	225492.25	225492.25	225492.25	225492.25	225492.25
Energy Gain [kWh/m²]	256.47	234.4	318.61	241.55	309.85	237.23	298.68	241.18
Energy Loss [kWh/m²]	-264.24	-240.51	-326.28	-247.59	-317.51	-243.29	-306.35	-247.15
System (Gain) [kWh/m²]	31.82	24.67	21.49	25.41	12.54	20.03	25	26.06
System (Loss) [kWh/m²]	-97.37	-83.86	-128.75	-88.79	-111.95	-84.64	-118.95	-87.92
Mech. Vent (Gain) [kWh/m²]	12.71	12.74	12.64	12.72	12.78	12.77	12.63	12.73
Mech. Vent (Loss) [kWh/m²]	-99.64	-100.22	-110.76	-100.63	-118.67	-102.52	-106.71	-100.26
Inf (Loss) [kWh/m²]	-33.25	-33.29	-34.38	-33.35	-35.01	-33.53	-33.94	-33.3
Inf (Gain) [kWh/m²]	10.92	10.91	10.89	10.91	10.87	10.89	10.89	10.91
Env (Gain) [kWh/m²]	13.76	15.01	37.24	16.16	35.51	15.79	29.25	15.98
Env (Loss) [kWh/m²]	-26.01	-21.83	-44.28	-20.88	-43.56	-21.38	-38.61	-21.42
Window (Gain) [kWh/m²]	24.53	7.86	73.77	13.17	75.42	14.39	58.12	9.9
Window (Loss) [kWh/m²]	-7.96	-1.32	-8.11	-3.94	-8.31	-1.23	-8.14	-4.25
Fan (Gain) [kWh/m²]	0	0	0	0	0	0	0	0
Lighting (Gain) [kWh/m²]	25.55	26.03	25.38	26	25.54	26.19	25.6	28.41
People (Gain) [kWh/m²]	137.18	137.18	137.18	137.18	137.18	137.18	137.18	137.18
Location [EPW Loc]	Erbil.Intl.AP	Erbil.Intl.AP	Erbil.Intl.AP	Erbil.Intl.AP	Erbil.Intl.AP	Erbil.Intl.AP	Erbil.Intl.AP	Erbil.Intl.AP
Opaque Envelope Area [m²]	2292.38	2200.5	2292.38	2200.5	2292.38	2200.5	2292.38	2200.5
Facade Area [m²]	1379.88	1288	1379.88	1288	1379.88	1288	1379.88	1288
Roof Area [m²]	456.25	456.25	456.25	456.25	456.25	456.25	456.25	456.25
Ext Facade Area [m²]	456.25	456.25	456.25	456.25	456.25	456.25	456.25	456.25
Ground Area [m²]	0	0	0	0	0	0	0	0
Ground Wall Area [m²]	0	0	0	0	0	0	0	0
Floor Area [m²]	1643.75	1643.75	1643.75	1643.75	1643.75	1643.75	1643.75	1643.75
Partition Area [m²]	0	0	0	0	0	0	0	0
Window Area [m²]	153.12	245	153.12	245	153.12	245	153.12	245
North Window Area [m²]	153.12	245	0	0	0	0	0	0
East Window Area [m²]	0	0	153.12	245	0	0	0	0
South Window Area [m²]	0	0	0	0	153.12	245	0	0

Table continue

	lorth Simulation R	Opt.North	East Simulation R3	Opt.East	outh Simulation R	Opt.South	Vest Simulation R3	Opt.West
West Window Area [m²]	0	0	0	0	0	0	▲ 153.12	▲ 245
Up Window Area [m²]	0	0	0	0	0	0	0	0
Ceiling Area [m²]	1187.5	1187.5	1187.5	1187.5	1187.5	1187.5	1187.5	1187.5
Envelope U-Val [W/(m²·K)]	1.99	▼ 1.95	1.99	▼ 1.95	1.99	▼ 1.95	1.99	▼ 1.95
Facade U-Val [W/(m²·K)]	3.13	3.13	3.13	3.13	3.13	3.13	3.13	3.13
Roof U-Val [W/(m²·K)]	0.16	0.16	0.16	0.16	0.16	0.16	0.16	0.16
Ext Floor U-Val [W/(m²·K)]	0.39	0.39	0.39	0.39	0.39	0.39	0.39	0.39
Ground U-Val [W/(m²·K)]	0	0	0	0	0	0	0	0
Ground Wall U-Val [W/(m²·K)]	0	0	0	0	0	0	0	0
Partition HC [kJ/m²]	0	0	0	0	0	0	0	0
Floor HC [kJ/m²]	338.79	338.79	338.79	338.79	338.79	338.79	338.79	338.79
Window U-Val [W/(m²·K)]	3.51	▼ 0.58	3.51	▼ 1.26	3.51	▼ 0.58	3.51	▼ 1.26
Window SHGC	0.74	▼ 0.16	0.74	▼ 0.11	0.74	▼ 0.16	0.74	▼ 0.11
Window TVis	0.88	▼ 0.29	0.88	▼ 0.07	0.88	▼ 0.29	0.88	▼ 0.07
Has Dynamic Shading	0	0	0	0	0	0	0	0
Envelope R-Val [W/(m²·K)]	1.82	▲ 1.89	1.82	▲ 1.89	1.82	▲ 1.89	1.82	▲ 1.89
People Density [p/m²]	0.3	0.3	0.3	0.3	0.3	0.3	0.3	0.3
Equipment Power Density [W/m²]	2	2	2	2	2	2	2	2
Lighting Power Density [W/m²]	16	16	16	16	16	16	16	16
Occupancy Sched Load Hrs	3650	3650	3650	3650	3650	3650	3650	3650
Equip Avail Sched Load Hrs	4161	4161	4161	4161	4161	4161	4161	4161
Lights Avail Sched Load Hrs	1825	1825	1825	1825	1825	1825	1825	1825
Dimming On [%]	1	1	1	1	1	1	1	1
Cooling COP	3	3	3	3	3	3	3	3
Heating COP	3	3	3	3	3	3	3	3
Heating Set Point [C]	21.01	21.01	21.01	21.01	21.01	21.01	21.01	21.01
Cooling Set Point [C]	26.02	26.02	26.02	26.02	26.02	26.02	26.02	26.02
Min Fresh Air Person [L/s/p]	10	10	10	10	10	10	10	10
Min Fresh Air Area [L/s/m²]	1	1	1	1	1	1	1	1
Has Heating [%]	1	1	1	1	1	1	1	1
Has Cooling [%]	1	1	1	1	1	1	1	1
Has Mech Vent [%]	1	1	1	1	1	1	1	1
Has Heat Recovery [%]	1	1	1	1	1	1	1	1
Heat Recovery Efficiency [%]	0.6	0.6	0.6	0.6	0.6	0.6	0.6	0.6
Infiltration [ACH]	0.5	0.5	0.5	0.5	0.5	0.5	0.5	0.5
Dom Hot Water COP	3	3	3	3	3	3	3	3
Water Temperature Inlet [C]	10.01	10.01	10.01	10.01	10.01	10.01	10.01	10.01
Water Supply Temp [C]	60.04	60.04	60.04	60.04	60.04	60.04	60.04	60.04
Water Schedule Load Hours [h]	8760	8760	8760	8760	8760	8760	8760	8760
Water Flow Rate Per Person [m³/h/P]	0	0	0	0	0	0	0	0
Has Dom Hot Water [%]	0	0	0	0	0	0	0	0

Supplementary Table S3 ClimateStudio outputs, a concise overview of the assessment process involving the utilization of double-skin facade systems. It includes a comparative analysis between a baseline model and an optimized model, highlighting their respective performance outputs with a focus on thermal energy insights.
